# Supplementary material for: BIN1 regulates actin-membrane interactions during IRSp53-dependent filopodia formation
Source: Commun Biol. 2024 May 9;7:549. doi: 10.1038/s42003-024-06168-8 (PMC11082164; doi:10.1038/s42003-024-06168-8)

SUPPLEMENTARY INFORMATION

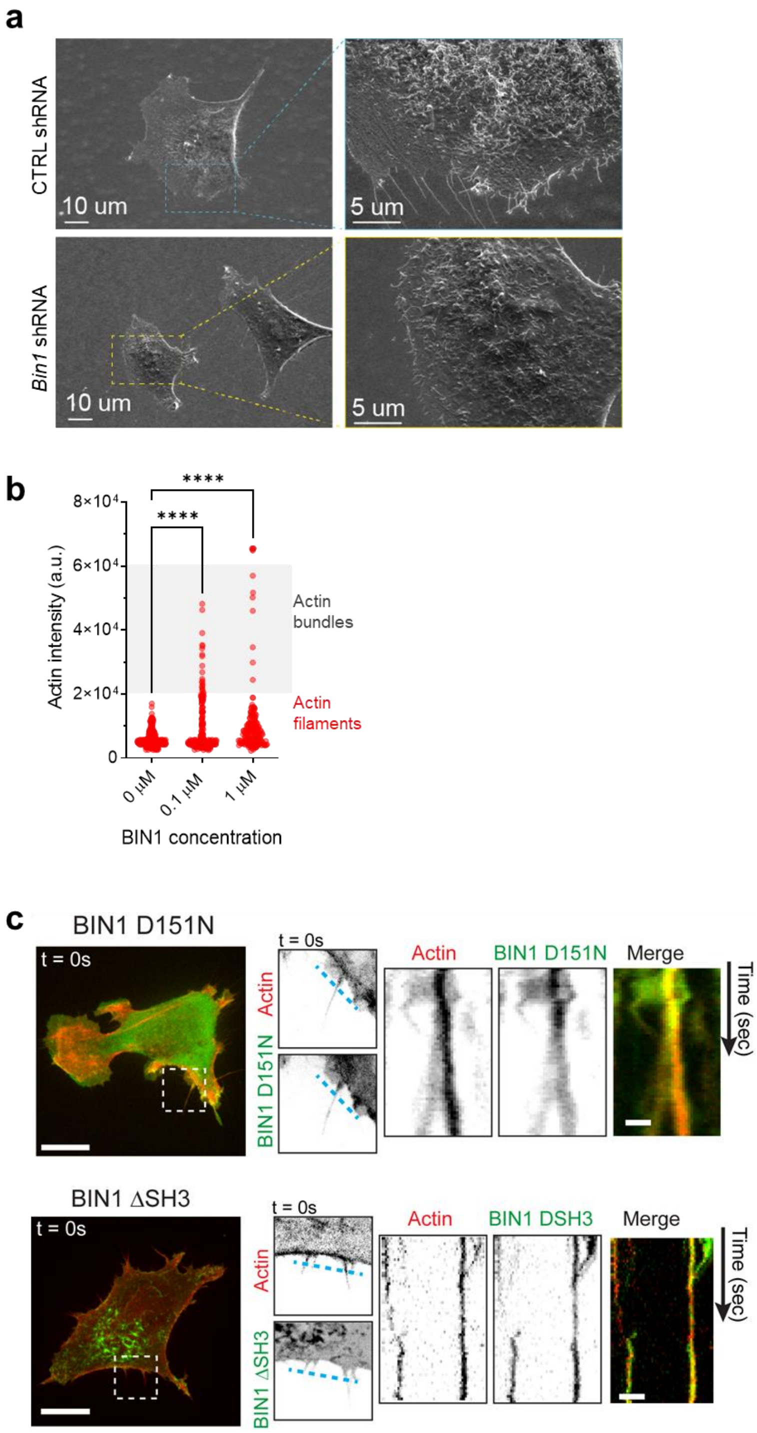

Supplementary Figure 1. a) Representative SEM images displaying the plasma membrane organization of isolated C2C12 myoblasts expressing either *Bin1* shRNA or CTRL shRNA (*luciferase*) under proliferative conditions. **b)** Quantification of the BIN1 bundling activity as denoted by the actin intensity in the absence (0 µM BIN1) or in the presence of BIN1 (0.1 µM BIN1 iso8 or 1 µM BIN1 iso8). **c)** Snap-shots of spinning disk images showing C2C12 cells co-transfected with either GFP-BIN1 D151N or GFP-BIN1 ΔSH3 mutant and Lifeact-mCherry (actin). Scale bar, 10 µm.

Protein	Accession number	Mascot Score	Unique Peptide
BIN1	O00499	1519	31
ACTIN	P60709	218	7
MOESIN	P26038	214	21
DYNAMIN-1	Q05193	207	8
EZRIN	P15311	191	10
IRSP53/BAIAP2	Q9UQB8	169	12
DYNAMIN-2	P50570	159	7
RADIXIN	P35241	65	2

Supplementary Figure 2. Identification of some binding partners of GFP-BIN1 using proteomic analysis. GFP alone was used as a negative control. Only Mascott Scores superior to 50 were considered as positive hits. In gray, already known BIN1 partners. In yellow, new identified BIN1 partners.

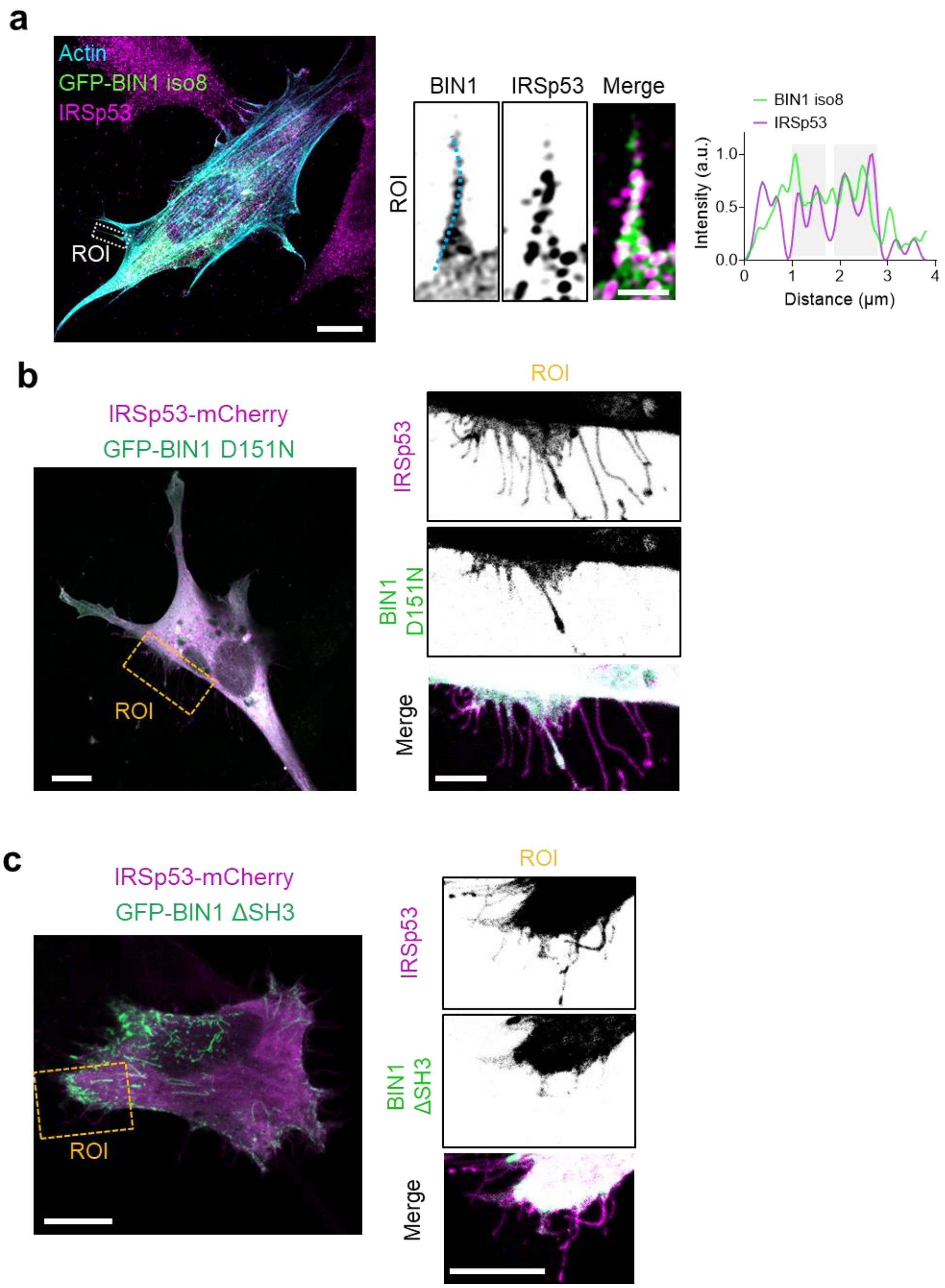

Supplementary Figure 3. a) Confocal images of C2C12 cells transfected with GFP-BIN1 iso8 (in green) and stained for endogenous IRSp53 (magenta) and F-actin (phalloidin, cyan). Magnified image of the corresponding ROI. Profile analysis of GFP-BIN1 iso8 (green) and IRSp53 (magenta) along the blue-dashed line in the ROI image. Scale bar, 10 µm. Scale bar ROI, 2 µm. **b-c)** Confocal images of C2C12 cells transfected with GFP-BIN1 D151N mutant (B) or GFP-BIN1 ΔSH3 (in green) and IRSp53-mCherry (magenta). Magnified image of the corresponding ROI. Scale bar, 10 µm. Scale bar ROI, 5 µm.

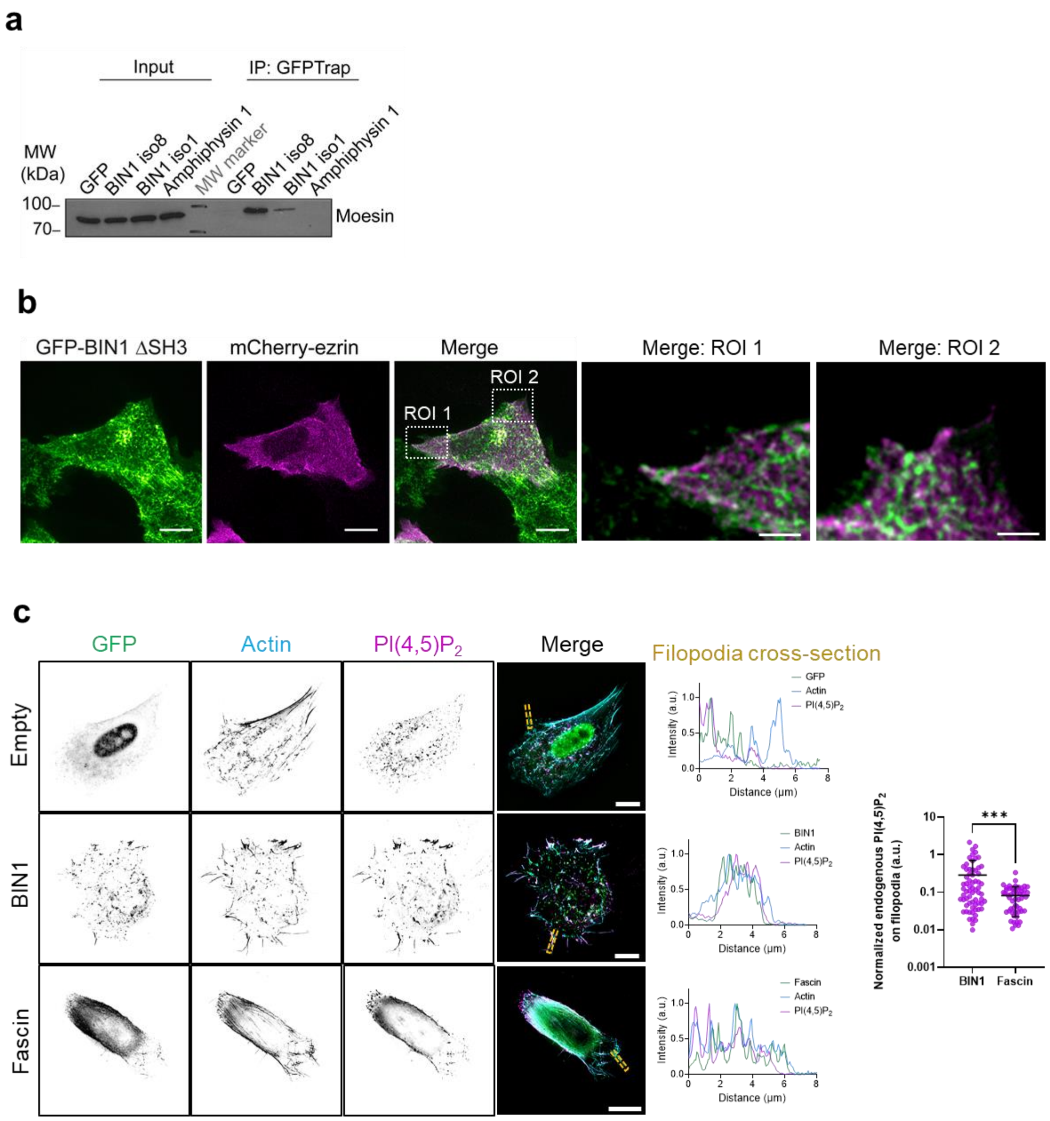

Supplementary Figure 4. a) GFP-Trap pull-downs from extracts of HeLa cells transfected with plasmids encoding GFP, GFP-tagged amphiphysin1, BIN1 iso1 and BIN1 iso8. Moesin was revealed by western-blotting. **b)** Time projection images from spinning disk movies (500 ms, 120 s) of C2C12 myoblasts co-expressing mCherry-ezrin (magenta) and GFP-BIN1 ΔSH3 (green). Scale bar, 10 µm. ROI scale bar, 2 µm. **c)** Wild-field deconvoluted images of C2C12 cells transfected with either GFP, GFP-BIN1 iso8 or GFP-Fascin and stained for endogenous PI(4,5)P_2 (PH-PLCd-Alexa647, magenta) and F-actin (phalloidin, cyan). Cross-section analysis of filopodia structures corresponding to the highlighted yellow region. Quantification of the normalized intensity of endogenous PI(4,5)P_2 (as the ratio between PI(4,5)P_2 intensity at filopodia respect to PI(4,5)P_2 intensity of the whole cell) at filopodia induced by BIN1 and Fascin, respectively. Scale bar, 10 µm.

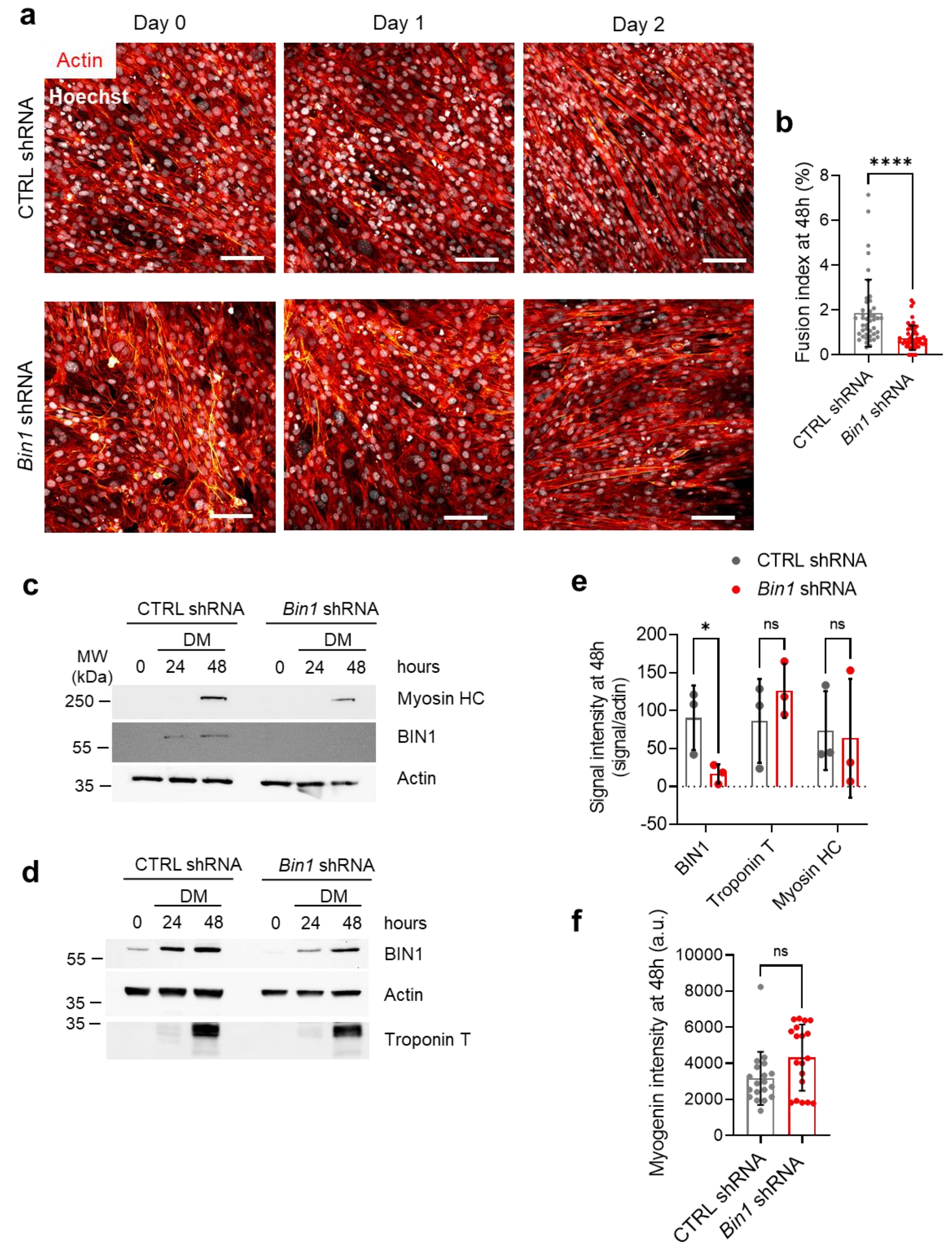

Supplementary Figure 5. a) Confocal images of C2C12 myoblasts CTRL shRNA or *Bin1* shRNA stained for F-actin (phalloidin, in red) and the nuclei (Hoechst, in gray). Scale bar = 100 µm. **b)** Quantification of the fusion index (number of nuclei in myotubes divided by the total number of nuclei) of the C2C12 CTRL shRNA (gray) and *Bin1* shRNA (red) at 48h in differentiation medium. Number of myotubes, n = 43 and 50, for CTRL shRNA and *Bin1* shRNA, respectively. **c, d)** Western-blot analysis of the endogenous expression of actin, BIN1, myosin heavy-chain (HC), and Troponin T in CTRL shRNA and *Bin1* shRNA C2C12 myoblasts in growth medium (0 hours, undifferentiated) and grown in differentiation medium (DM) at 24h and 48h. **e)** Quantification of the signal intensity (signal/actin) of the endogenous expression of BIN1, Troponin T and myosin HC in CTRL shRNA and *Bin1* shRNA

C2C12 myoblasts grown in DM for 48h obtained from western-blot analysis. **f)** Quantification of the intensity of the endogenous expression of myogenin in CTRL shRNA and *Bin1* shRNA C2C12 myoblasts grown in DM for 48h from confocal images (immunofluorescence). Number of images, n = 19 and 19, for CTRL shRNA and *Bin1* shRNA, respectively Mann Whitney test: n.s > 0.1, * P < 0.05., **** P < 0.0001. All data represents three independent experiments (N = 3).

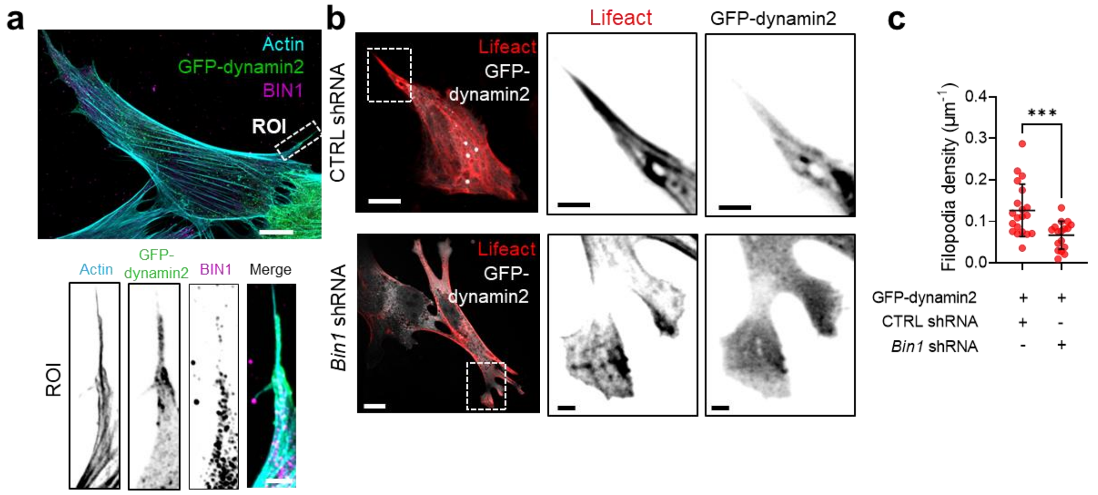

Supplementary Figure 6. a) Confocal/airyscan images of C2C12 cells transfected with GFP-dynamin2 (in green) and stained for endogenous BIN1 (magenta) and F-actin (phalloidin, cyan). Magnified image of the corresponding ROI. Scale bar, 10 µm. Scale bar ROI, 2 µm. **b)** Z-projected confocal images showing the actin organization of CTRL shRNA and *Bin1* shRNA C2C12 cells co-transfected with GFP-dynamin2 (gray) and Lifeact-mCherry (red). **c)** Quantification of filopodia density; number of cells, *n*=20 and 17 for CTRL shRNA and *Bin1* shRNA C2C12 cells co-transfected with GFP-dynamin2 and Lifeact-mCherry respectively. Error bars represent s.d.; t-test: *** P < 0.001.

Supplementary Figure 7

Figure 1b

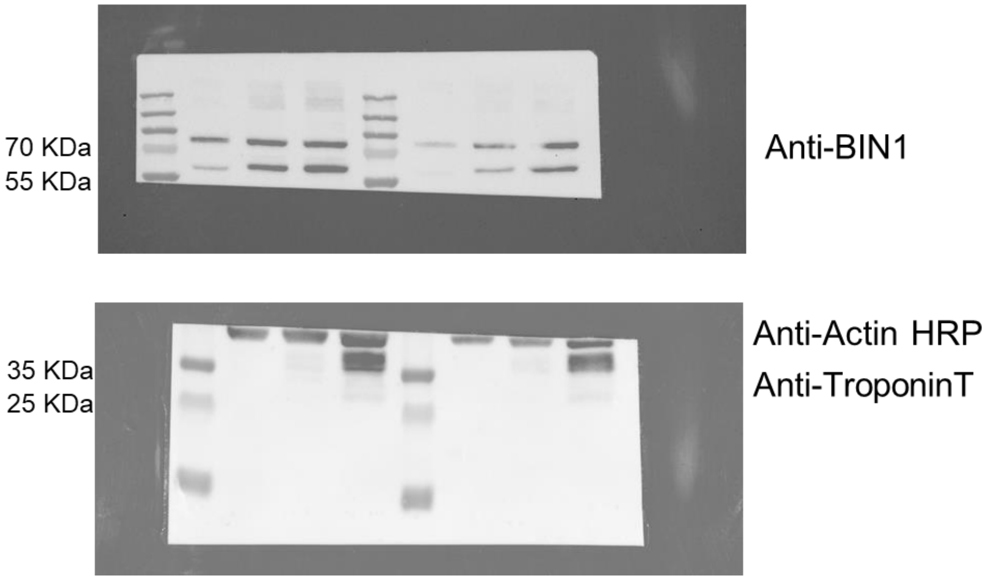

Figure 1c

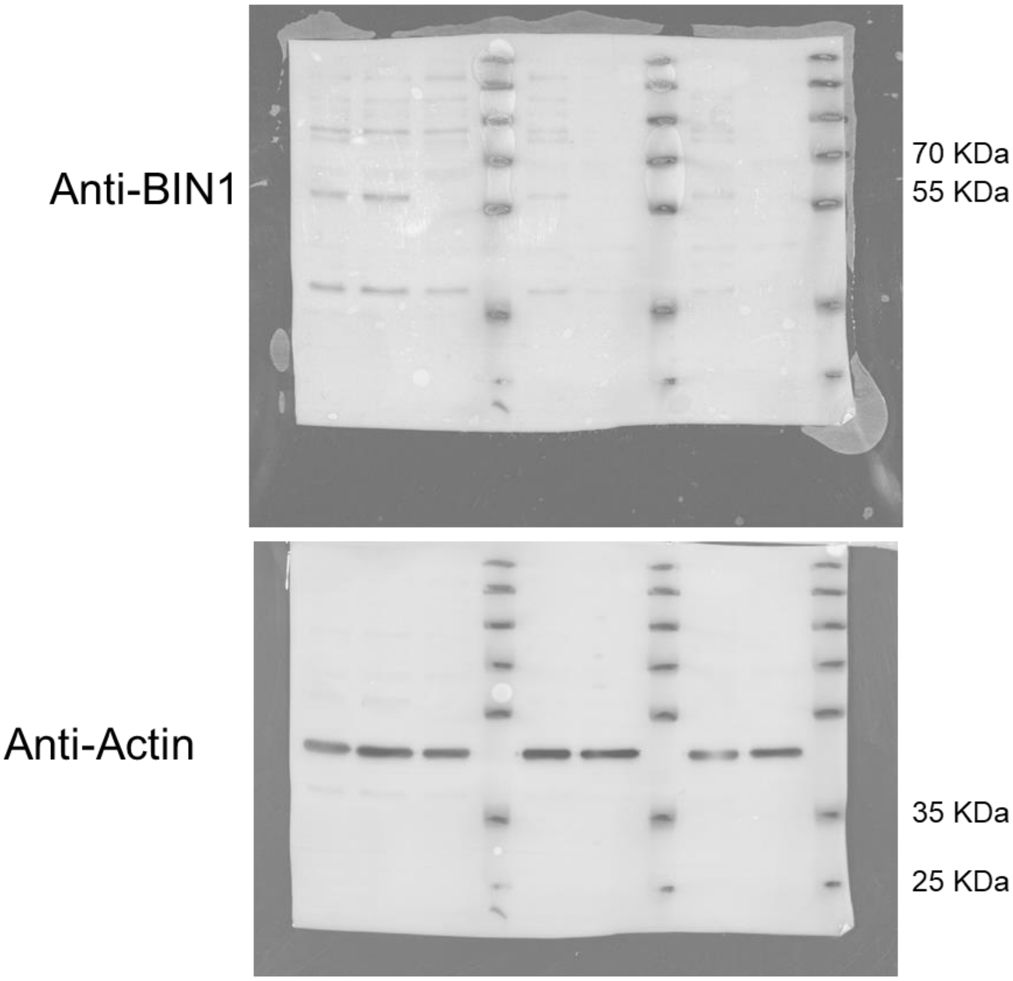

Figure 2c

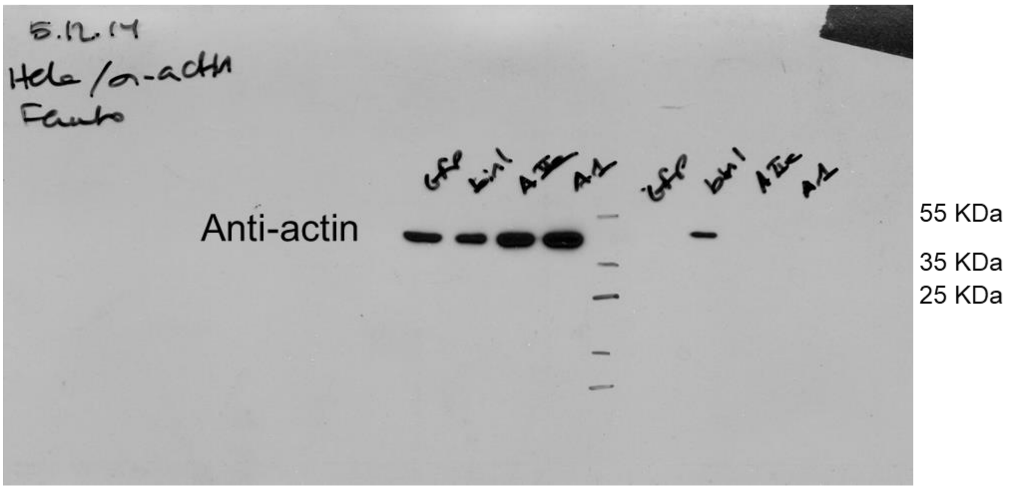

Figure 2e

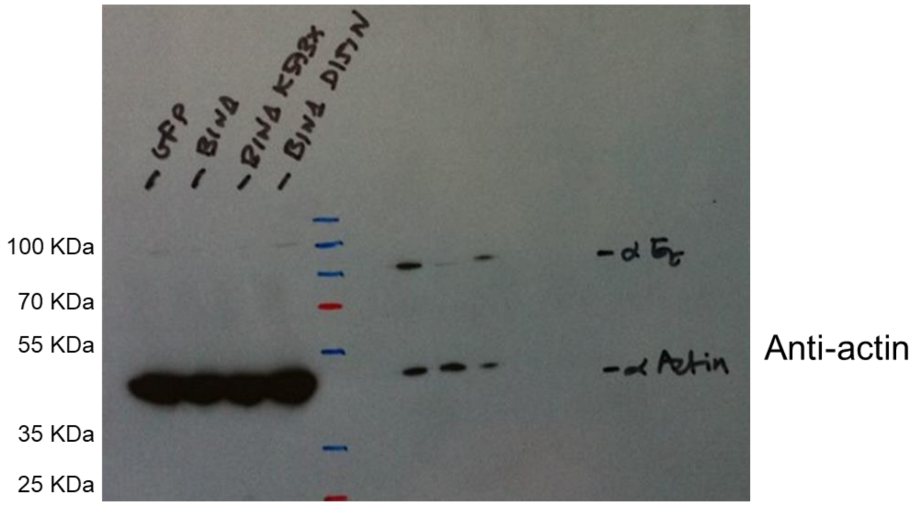

Figure 3c

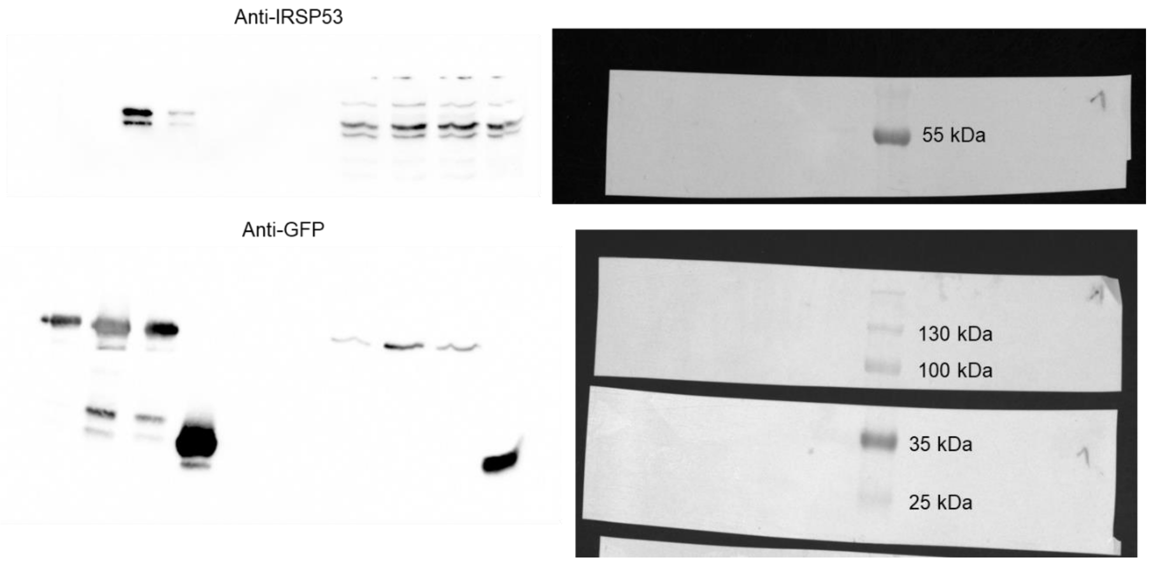

Figure 3d

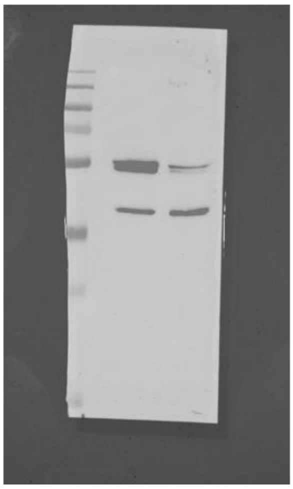

Figure 4a

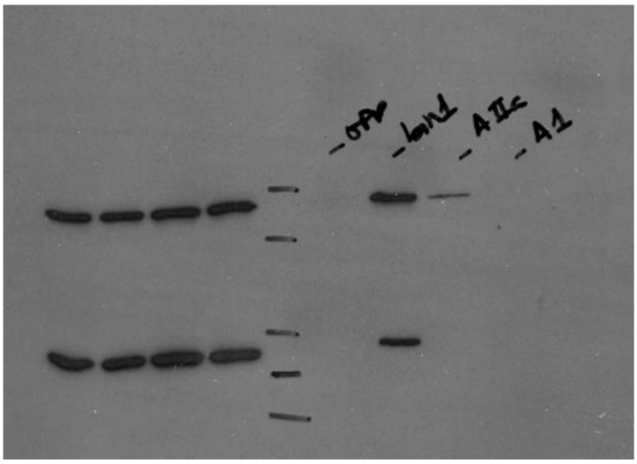

Figure 4b

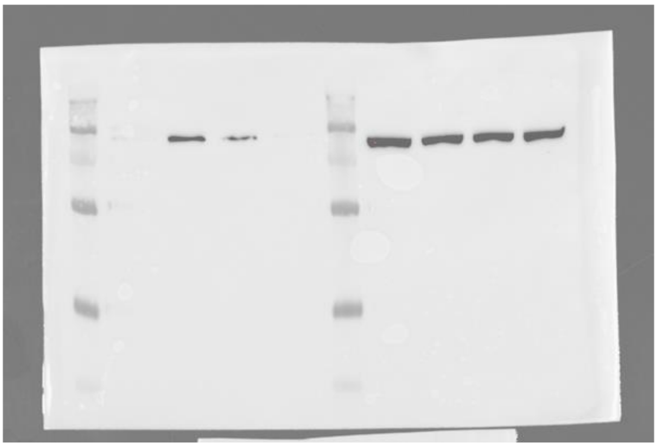

Figure 5a

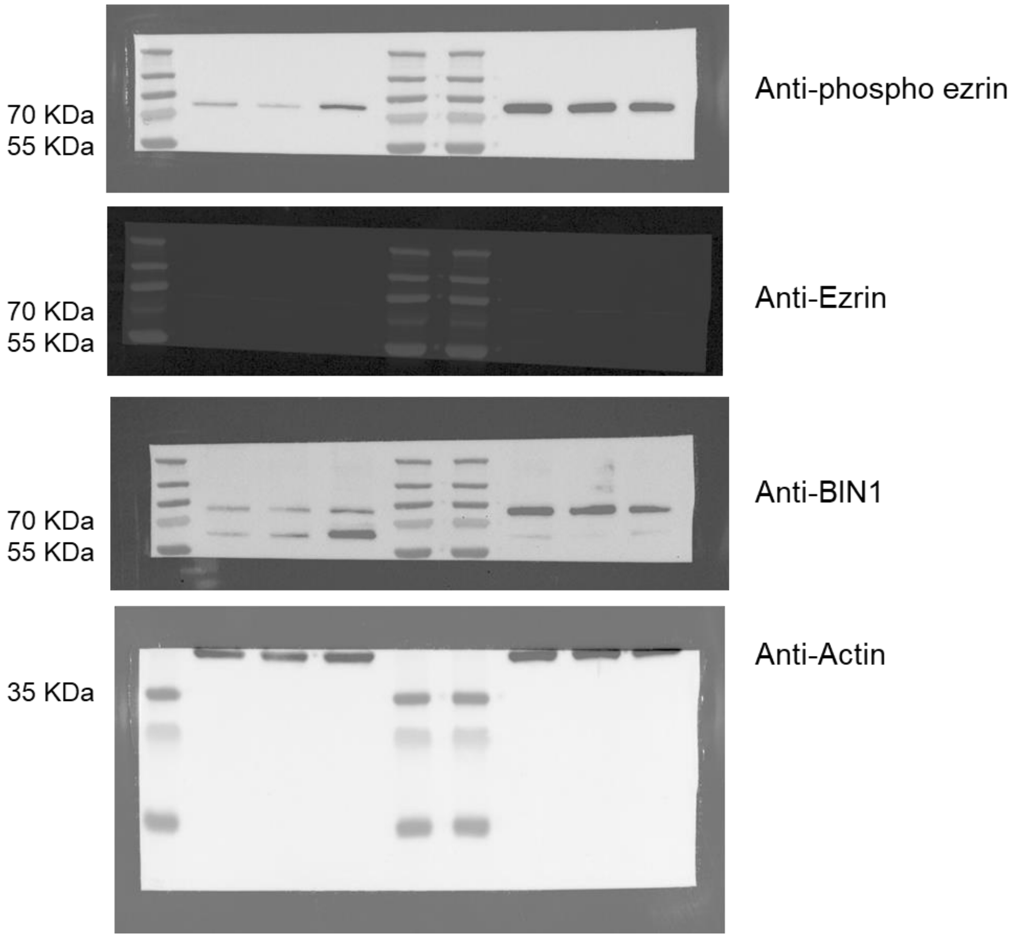

Supplamentary Figure 4

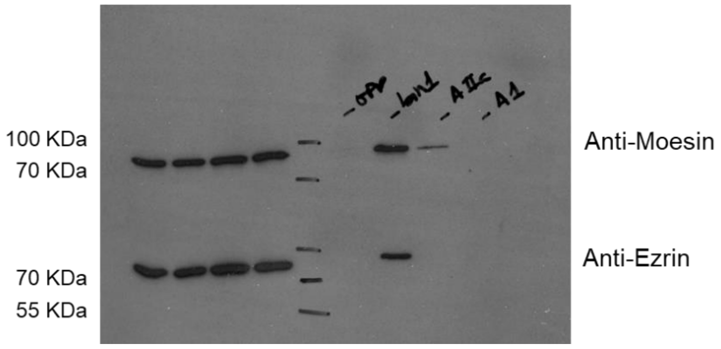

Supplamentary Figure 5c

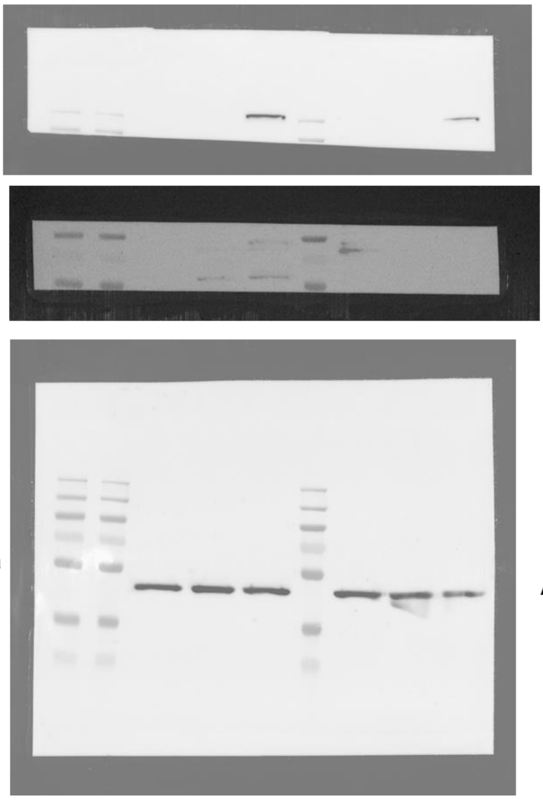

Supplamentary Figure 5d

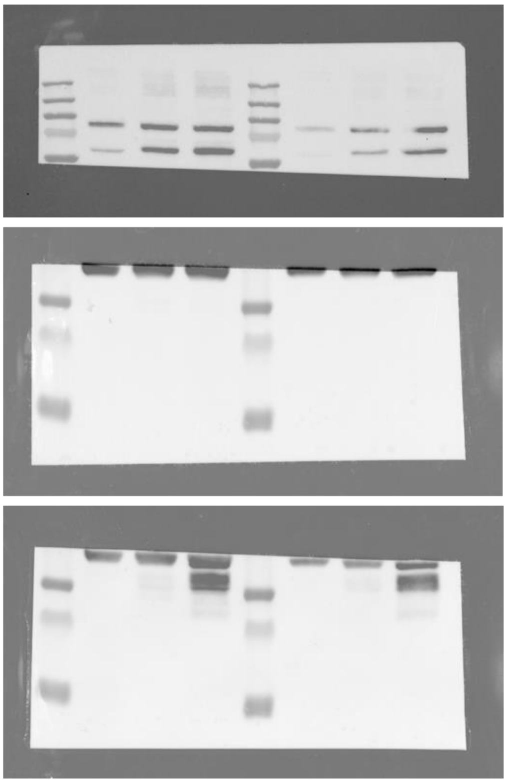

Supplement: Supplementary file 2 — Supplementary information [file 42003_2024_6168_MOESM2_ESM.pdf]
