# Supplementary material for: BIN1 regulates actin-membrane interactions during IRSp53-dependent filopodia formation
Source: Commun Biol. 2024 May 9;7:549. doi: 10.1038/s42003-024-06168-8 (PMC11082164; doi:10.1038/s42003-024-06168-8)
Supplement: Supplementary file 3 — Description of Additional Supplementary Files [file 42003_2024_6168_MOESM3_ESM.pdf]

Description of Additional Supplementary Files

File name: Supplementary Data 1

Description: Source data behind the graphs in the paper is presented in separate tabs of the Excel file.

File name: Supplementary Data 2

Description: Mass spectrometry-based proteomics data
